# The Immune Privilege of the Intervertebral Disc: Implications for Intervertebral Disc Degeneration Treatment

**DOI:** 10.7150/ijms.42238

**Published:** 2020-02-24

**Authors:** Zhen Sun, Bing Liu, Zhuo-Jing Luo

**Affiliations:** 1 Department of Orthopedic, Xijing Hospital, Fourth Military Medical University. Western Changle Road, Xi'an, 710032, Shannxi Provence, P. R. China.; 2 Department of Radiology, Xijing Hospital, Fourth Military Medical University. Western Changle Road, Xi'an, 710032, Shannxi Provence, P. R. China.

**Keywords:** intervertebral disc, nucleus pulposus, annulus fibrosus, immune privilege, auto-immune response

## Abstract

The intervertebral disc (IVD) is the largest avascular organ of the body. It is composed of three parts: the nucleus pulposus (NP), the annulus fibrosus (AF) and the cartilaginous endplate (CEP). The central NP is surrounded by the AF and sandwiched by the two CEPs ever since its formation. This unique structure isolates the NP from the immune system of the host. Additionally, molecular factors expressed in IVD have been shown inhibitive effect on immune cells and cytokines infiltration. Therefore, the IVD has been identified as an immune privilege organ. The steady state of immune privilege is fundamental to the homeostasis of the IVD. The AF and the CEP, along with the immunosuppressive molecular factors are defined as the blood-NP barrier (BNB), which establishes a strong barrier to isolate the NP from the host immune system. When the BNB is damaged, the auto-immune response of the NP occurs with various downstream cascade reactions. This effect plays an important role in the whole process of IVD degeneration and related complications, such as herniation, sciatica and spontaneous herniated NP regression. Taken together, an enhanced understanding of the immune privilege of the IVD could provide new targets for the treatment of symptomatic IVD disease. However, the underlying mechanism above is still not fully clarified. Accordingly, the current study will extensively review and discuss studies regarding the immune privilege of the IVD.

## Introduction

Intervertebral disc (IVD) degeneration is one of the most common contributors to spinal degenerative disease, which leads to individual sufferings such as acute/chronic pain, disability and psychological problems, causing enormous social and economic burden 1, 2. Current strategies for IVD degeneration management include conservative and surgical treatment 3. Recently, biological options, such as biomaterials application, cell translation and genetic modification, have shown promising beneficial effects on IVD regeneration 4-7. However, the pathological process of IVD degeneration is still not fully understood.

As the largest avascular organ of the body, the IVD sits between the vertebras and is responsible for the support, durability and flexibility of the spine 8. Anatomically, the IVD is a complex tissue comprising of three parts: a central proteoglycan-rich core, the nucleus pulposus (NP), an outer circumferential ring of fibrocartilage, the annulus fibrosus (AF), and the two cartilaginous endplates (CEP) adjoining the vertebra bodies 9. The gelatinous NP is composed of cell clusters embedded in a proteoglycan-collagen-rich extracellular matrix (ECM). Ever since its formation, the NP tissue has been trapped in the IVD by the surrounding AF and CEP, and this unique structure isolates the NP from the immune system of the host. Meanwhile, various ingredients of the NP were found to cause auto-immune reactions after exposure to the host immune system during IVD degeneration progress 10, 11. For this reason, the IVD has been identified as an immune privilege organ. In fact, these features have long been observed and the concept of IVD immune privilege has been proposed. Although the immune privilege status is essential for IVD homeostasis and normal function, there have been no studies summarizing the state-of-the-art in this field until now. Therefore, the current study will extensively review and discuss relative studies regarding the immune privilege of the IVD.

### The development of the IVD

**Figure 1:**
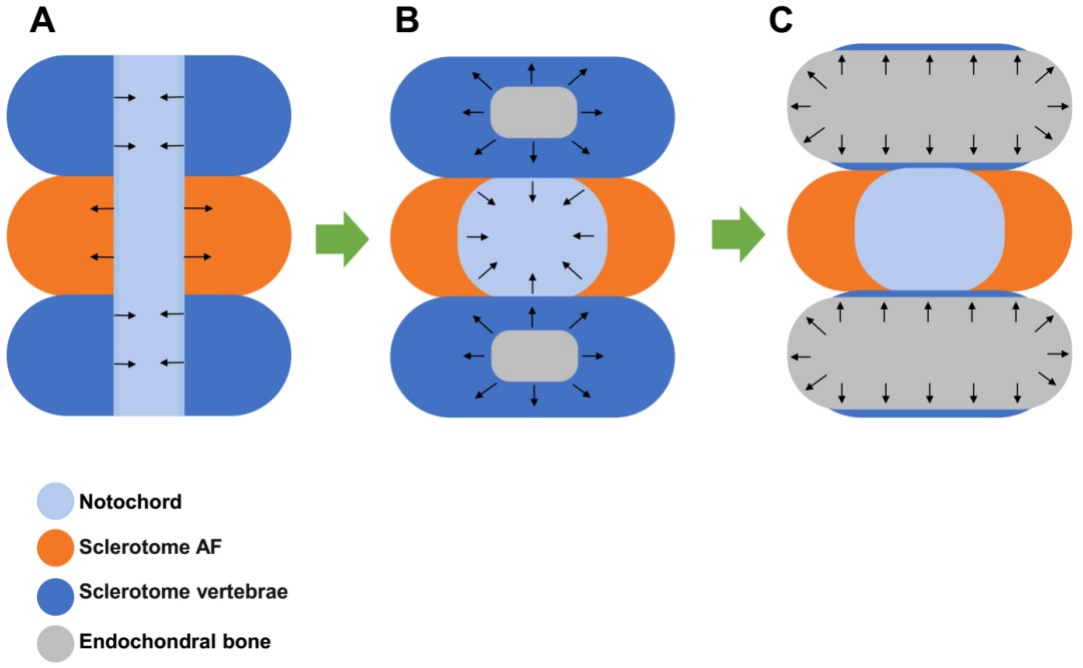
Development of the intervertebral disc (IVD). **A**. Sclerotome condenses around the notochord to form the vertebrae and the putative annulus fibrosus (AF) while the notochordal is contracted from the vertebral body and expands into the area of the future nucleus pulposus (NP) area. **B**. The notochordal is compressed and becomes entrapped in the surrounding dense ring of sclerotome-derived connective tissue, which develops to the AF. **C**. Endochondral bone formation occurs in the sclerotome vertebra and expands to become bony vertebra. Hyaline cartilage adjacent to the IVD is maintained and develops to the cartilaginous endplate (CEP).

The IVD is derived from embryonic structures termed as the sclerotome and notochord between the developing vertebrae. With the formation of vertebrae, sclerotome condenses around the notochord to form the vertebrae and the putative AF. At the same time, notochordal is contracted from the vertebral body and expands into the area of the future NP. The notochordal is compressed and become entrapped in a dense ring of sclerotome-derived connective tissue. The centralized notochordal element develops to the NP tissue in the early fetal life and grows rapidly in the late fetal life and early infancy. On the other hand, the surrounding dense ring of sclerotome-derived connective tissue develops to the AF with abundant ECM containing collagens and glycoproteins. Meanwhile, chondrocyte differentiation and endochondral bone formation occur in the vertebral bodies with high concentrations of bone morphogenetic protein (BMP) activity 12. When the bony vertebra is formed, hyaline cartilage adjacent to the IVD is maintained and develops to the CEP at the end plate (Figure 1). In the early stage of human life, the NP is populated by clusters of large, vacuolated notochordal cells and by small chondrocyte-like cells 13. However, by the second decade of life, the notochordal cells in the NP disappear, and the NP transitions from a notochordal structure to a tissue embedded with small chondrocyte-like cells 14. It is speculated that early IVD degeneration happens with the disappearance of notochordal cells 15. During this process, it is noteworthy that the NP tissue is sealed and isolated from the immune system ever since its formation at the beginning of IVD development.

### The blood-NP barrier

Immune privilege organs are operationally defined as regions in the body where foreign tissue grafts can survive and extend with indefinite periods of time, while similar implants placed at regular regions of the body are acutely rejected. Well studied structures include the eye 16 and the brain 17, along with the testis 18 and pregnant uterus 19. In these structures, barriers between the internal environment and the host immune system have been widely observed and explored in-depth. However, the mechanism of NP-host immune system isolation is still undefined.

Accumulating evidence has suggested the existence of machinery that limits immunocytes and immune mediators entering the NP tissue in IVD. This machinery, here, could be defined as a blood-NP barrier (BNB), which is a complex composition of physical and molecular factors. In anatomical view, the BNB is a region that isolates the NP tissue from the host immune system. It is generally composed of the AF and the CEP. The AF is composed of 15-25 concentric layers, which consists of alternatingly aligned oblique collagen fibers interspersed with proteoglycans. This feature holds the NP by confine its swelling pressure and resisting shear and tensile stresses from the internal pressure exerted by the NP 12. The CEP is a layer of cartilage with about 0.5-1 mm thickness. It contains ECM of aggrecan and collagens with chondrocytes embedded in it 20. The CEP serves not just as an interface between the soft NP and the dense bone of the vertebrae, but as a physical barrier that prevents immune cells and cytokines to the bone. In addition, studies have shown that the high proteoglycan concentration, together with micro-environment of high physical pressure, inhibits the ingrowth of blood vessels 21, which act as significant channel of immunocytes infiltration. Therefore, the AF and the CEP constitute a strong basement isolating the NP tissue from the host immune system.

On the other hand, pilot studies have found the inhibition effect of molecular factors expressed in IVD and indicated their important role in the long-term maintenance of IVD immune privilege. Studies showed that Fas ligand (FasL), which is an apoptosis inducer and widely expressed in other immune privilege sites, exists in human NP tissues 22, 23. In our previous studies, we found that FasL could induce apoptosis of both vascular endothelial cells and immunocytes including macrophages and CD8^+^T cells 24, 25. These studies indicate that FasL might act as a molecular barrier by eliminating blood vessel infiltration and immune cells recruitment. In another study, Wiet et al. found that healthy AF conditioned medium obtained from AF cultures could inhibit mast cell activation by downregulating its expression of vascular endothelial growth factor (VEGF), tumor necrosis factor (TNF)-α, interleukin (IL)-1β and chemokine (C-C motif) ligand 2 (CCL2/MCP-1), and inhibit mast cell induced angiogenesis 26. In addition, numerous studies have reported the protective effect of notochordal cells in IVD and its suppressive impact on inflammation 27, 28. Cornejo et al. and Kwon et al. found soluble factors from notochordal cells inhibit endothelial cell invasion and vessel formation by suppressing VEGF signaling 29, 30. Meanwhile, Purmessur et al. found intact glycosaminoglycans from IVD-derived notochordal cell-conditioned media inhibit neurite growth while maintaining neuronal cell viability 31. Yet, in the study of de Vries et al., the anti-angiogenic and anti-neurogenic effects of notochordal cell-conditioned media were not observed 32. This is, as the authors pointed, attributed to the different life stages and breeds of sacrificed animals, and cell culture conditions. However, evidence is still limited in the interaction between notochordal cells and immunocytes, and the role of notochordal cells in IVD immune privilege needs more studies.

**Figure 2:**
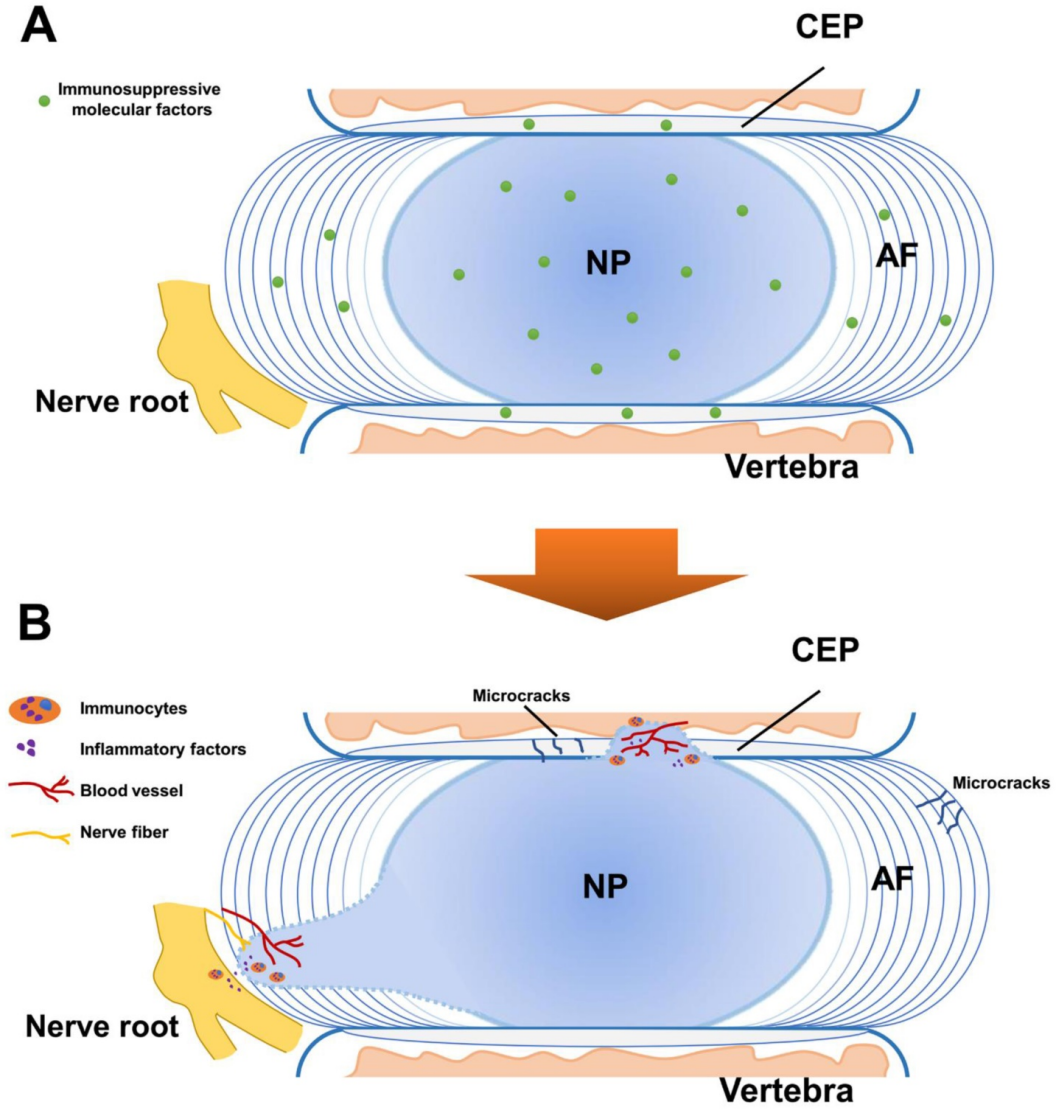
Schematic representation for the immune privilege of the intervertebral disc (IVD). **A**. In normal IVD, the blood-NP barrier (BNB) is composed of the annulus fibrosus (AF), the cartilaginous endplate (CEP) and immunosuppressive molecular factors. The BNB isolates the central nucleus pulposus (NP) from the immune system of the host and provides fundamental basis for the homeostasis of the IVD. **B**. The breakdown of the BNB leads to the exposure of the NP and induces auto-immune response. This effect causes immunocytes activation and inflammatory factors infiltration, contributing to the immune stress of the nerve root with vascularization and neurotization.

Collectively, current studies are still limited in the immunosuppressive effects of molecular factors in IVD. The exact molecules mediating these effects are still not fully understood. Multiple factors such as exosomes and miRNAs might be involved and more studies are needed to clarify the mechanism in this aspect. Altogether, the AF, the CEP and molecular factors such as FasL, establish a unique architecture for immune privilege resembling that of a medieval castle (Figure 2A).

### Auto-immune response of the NP

In IVD degeneration, BNB damage such as fissure and tear is commonly observed as pathological change in the AF and CEP 33, 34. It has been shown that fissure of AF is mechanically and chemically conducive to the ingrowth of blood vessels 35. The auto-immune response and downstream cascade reaction starts when the BNB is damaged. In fact, as early as the 1960s, studies have found the evidence of auto-immune response of the degenerated NP in patients and animals 36, 37, and indicated that radicular pain of a lumbar disc herniation results from chemicals of exposure of the NP and related auto-immune response 38. Following studies were conducted trying to explore this phenomenon. In particular, Satoh et al. studied eight patients with lumbar disc herniation and found antigen-antibody complexes seem to be commonly present in the herniated NP tissue 39. To identify the immunocyte types, Geiss A et al. found activated T and B cells were elevated by autologous NP subcutaneously in an animal model 40. In another study, they found that T cells could be activated by autologous NP tissue simulation 41. Murai et al. found that macrophages and NK cells could recognize autologous NP cells and showed positive cytotoxic effects by comparation of wild type mice and immune-deficient mice 42. Moreover, Geiss A et al. found predominately plasmacytoid dendritic cells (PDCs) along with few macrophages and memory T cells in both sequestrated and extruded discs, suggesting that (PDCs) play an important role in initiation of an immune response of NP while macrophages may mediate disc resorption at a later stage 43. Most recently, Lee et al. showed macrophage infiltration into injured IVD along with pathological innervation in long-term histological analysis of punctured mice IVD model 44. In canine IVD herniation, Monchaux et al. found that monocytes and macrophages existed in extruded IVD material 45. These findings suggested complicated types of immunocytes involve in the auto-immune response of the NP in different stages and macrophages might play an important role in this pathological process. More studies are needed to explore the underly mechanism of their roles.

The direct evidence of the NP auto-immune response was found by Capossela et al., who identified IgGs against collagen type I, II, V, and aggrecan in human degenerated IVD samples 10. Moreover, inflammatory factors were found to have increased expression in the study of Takada et al, who found IVD autografts induced TNFα, IL-6 and IL-8; as well as cyclooxygenase 2 up-regulation and macrophage infiltration in sciatica 46. By co-culture of autologous or allogeneic peripheral blood mononuclear cells and NP cells, Stich et al. found elevated immune cell proliferation levels in 3D-cultures than 2D-cultures, and a general trend to higher responses for NP cells from severely degenerated IVD tissue 47, indicating that auto-immune response could vary depending on different cell cultures and degeneration degrees. More recently, Silva et al. established a model of IVD organ culture, found that human macrophages could be polarized toward a more pro-inflammatory profile by degenerated IVD tissue, and interfere with IVD ECM remodeling by downregulating aggrecan and collagen II gene expression in the presence of IL-1β 48. These findings showed that with the breakdown of the BNB, the exposed NP tissue could induce auto-immune response, which stimulates both immunocytes activation and inflammatory factors infiltration (Figure 2B).

Meanwhile, the recruitment of immunocytes could lead to the deterioration of IVD degeneration via cell-cell biocommunication and cytokines secretion. As for the IVD cells, studies have showed altered phenotypes and function in various auto-immune reactions. Ni et al. found that M1-polarized macrophages promote degenerative phenotypes in NP cells with increased expression of key matrix catabolic genes, reduced the expression of major matrix-associated anabolic genes and upregulated transcription of inflammation-related genes 49. Also, in a co-cultures system, Yang et al. showed that AF or NP cells exposed to macrophages upregulated the expression of pro-inflammatory mediators 50. As for the ECM, studies have found that immunocytes could interfere with IVD ECM remodeling and downregulate aggrecan and collagen II expression 48. In addition, the expression of matrix metalloproteinases (MMPs) and a disintegrin and metalloproteinase with thrombospondin motifs (ADAMTSs) were increased in IVD with auto-immune reaction, causing the degradation of ECM 51, 52. Taken together, the auto-immune reaction could stimulate immunocytes and inflammatory cytokines infiltration, and these factors could in turn impact on the IVD with harmful influence. Nevertheless, more studies are desired to explore the underlying mechanism and downstream pathways in the auto-immune response of NP tissue.

### Implications for clinical management of IVD degeneration

The steady state of IVD immune privilege is fundamental to the homeostasis of the IVD. The AF and the CEP, along with the immunosuppressive molecular factors, being regarded as the BNB, establish a strong barrier segregating the immune system from healthy IVD. However, the breakdown of the immune privilege could lead to profound consequences in IVD degeneration.

In the early stage of IVD degeneration, the breakdown of the BNB and the auto-immune reactions could act as a trigger with the downstream cascade reactions accelerating the pathological progress. Signs of BNB damage and NP exposure could be seen in Magnetic Resonance Imaging in early IVD degeneration. It has been indicated that Schmorl's nodes are the early stage of auto-immune response of the degenerated IVD 53. Also, type 1 Modic change, which is widely observed in early IVD degeneration, is thought to be an auto-immune response with CEP damage 54. Indeed, these image manifestations are strong evidence for IVD degeneration prognostication and early management 55-57. Additionally, studies have found that inflammatory factors are upregulated in serum from IVD herniation patients. Weber et al. found that serum levels of IL-6 were significantly higher in subjects with LBP compared with control subjects 58. Wang et al. showed that the expression of IL-10 and IL-17 was elevated in peripheral blood sera of IVD degeneration patients 59. Most recently, Hasvik et al. found that up-regulation of circulating microRNA-17, which mediates macrophage activetion with increased TNF production, is associated with lumbar radicular pain following disc herniation 60. Therefore, studies in the IVD auto-immune response are essential for early IVD degeneration diagnosis and management.

In the late stage of IVD degeneration, pathological changes associated with BNB damage and NP exposures are very common. These alternations include AF disruption, NP herniation and sciatica 61. Besides physical compression, studies have strongly suggested that auto-immune reaction of the NP is a key mediator of radicular pain in IVD herniation 38, 42. Studies have showed various types of activated immunocytes and inflammatory factors were recruited in NP-nerve root area 62, 63. The immunocytes were identified including macrophages, T cells, B cells, NK cells and mast cells, and the inflammatory factors were detected such as phospholipase A2 64, leukotrienes 65, fibroblast growth factor 66, the IL family 66, tumor necrosis factors 67, matrix metalloproteinases 68, nitric oxide 69, substance P 70, monocyte chemoattractant protein-1 71, vascular endothelial growth factor 72 and nerve growth factor 73. These factors constitute a complicated region contributing to the immune stress of the nerve root. In addition, vascularization and neurotization aggravate this situation with blood channel infiltration and nerve sensitization 74. Therefore, strategies aiming for nerve root immunoregulation are proposed 75, 76. In our study, we hypothesized that molecular immunotherapy might be a potential option in the treatment strategies for IVD degeneration and herniation 77.

While BNB plays an important role in normal IVD function, it is notable that not all IVD degenerations are a result of BNB failure. In specific, AF tears could lead to abnormal mechanical distribution, NP leakage and nerve ingrowth of the IVD. Additionally, microfractures in endplates which could be the result of single event trauma or part of ageing where the EP calcifies and is more prone to microfractures and exposing NP tissue to blood supply 78.

Until now, it remains a controversial topic as to the consequence of auto-immune response of IVD. While most studies indicate auto-immune response in disc herniation could be a critical factor to induce radicular pain, some studies have suggested the close relationship of auto-immune response with spontaneous regression. Komori et al. found that the disappearance of herniated NP was seen frequently in exposure to the vascular supply 79. By examine the histological features of herniated NP tissue; Ikeda rt al. concluded that extruded or sequestrated disc was resorbed by phagocytes 80. These studies indicate that the auto-immune response could be beneficial in some cases with exposed NP absorption. The observation of spontaneous resorption of IVD herniation has been widely reported 81-84 and the incidence of spontaneous resorption of lumbar disc herniation is reported as high as more than 60% 85. Moreover, by addressing the molecular and cellular mechanisms involved in herniated NP regression, Cunha et al. concluded that inflammatory response could be regarded as a good prognostic indicator of spontaneous regression 86. These studies suggested the importance of auto-immune reaction in conservative treatment for IVD herniation before final surgical involvement. Therefore, it is very important to clarify its molecular mechanism. However, the role of auto-immune reaction in this phenomenon is still unclear.

As for biological treatment for IVD degeneration, the immune privilege of the IVD cannot be ignored in stem cell transplantation and biomaterial application. In fact, stem cell could play an essential role in immune privilege rebuilding via various pathways in IVD regeneration 87. In addition, strategies aiming to avoid auto-immune reaction should be considered in biomaterial application, as biomaterials used in IVD regeneration are often formulated to mimic IVD composition, and usually with foreign substance.

## Conclusion

The concept of IVD immune privilege arose from the apparent segregation from immune system of the body. The immune privilege state is one of the key factors to provide stable environment keeping homeostasis and normal function of the IVD. The BNB is accordingly proposed here as a complex structure, which is composed of the NP, the AF and molecular factors, and limits the penetration of the immune system. However, the breakdown of BNB could lead to auto-immune reaction of the IVD and downstream pathways. These effects play an important role in IVD degeneration process including inducement, acceleration and prognosis. Nevertheless, the study of IVD immune privilege is still limited and more studies are needed to explore the underlying mechanisms for its formation, maintenance and breakdown.

## Abbreviations

ADAMTSs: a disintegrin and metalloproteinase with thrombospondin motifs
AF: annulus fibrosus
BMP: bone morphogenetic protein
BNB: blood-NP barrier
CEP: cartilaginous endplate
ECM: extracellular matrix
FasL: Fas ligand
IL: interleukin
IVD: intervertebral disc
MMPs: matrix metalloproteinases
NP: nucleus pulposus
TNF: tumor necrosis factor
VEGF: vascular endothelial growth factor
